# Serum Neuron-Specific Enolase as a Prognostic Biomarker in Pediatric Convulsive Status Epilepticus: A Single-Center Retrospective Cohort Study

**DOI:** 10.3390/children13060820

**Published:** 2026-06-15

**Authors:** Merve Yavuz, Ibrahim Bingol

**Affiliations:** 1 Department of Pediatric Neurology, Gaziantep City Hospital, Gaziantep 27060, Turkey; 2 Department of Pediatric Intensive Care, Gaziantep City Hospital, Gaziantep 27060, Turkey; ibrahimbingol@gmail.com

**Keywords:** status epilepticus, neuron-specific enolase, children, prognosis, pediatric intensive care unit, biomarker, neurological outcome, calibration, bootstrap validation

## Abstract

**Highlights:**

**What are the main findings?**

- Serum neuron-specific enolase (NSE) measured within 48 h of PICU admission was independently associated with poor neurological outcome (adjusted OR 1.11 per μg/L; 95% CI 1.06–1.19; *p* = 0.001) and showed good discrimination for in-hospital mortality (AUC 0.885; 95% CI 0.79–0.96) in 132 children with convulsive status epilepticus. The association persisted in separate analyses restricted to mortality (*n* = 132) and to neurological deterioration among survivors (*n* = 114).
- An exploratory NSE cutoff of 25.7 μg/L, closely approximating the institutional upper reference limit (25.0 μg/L), identified high-risk patients with high specificity (97.2%, 95% CI 90.4–99.2) and PPV (93.3%, 95% CI 78.7–98.2) but limited sensitivity (46.7%, 95% CI 34.6–59.1), consistent with a rule-in rather than screening profile.

**What are the implications of the main findings?**

- These findings are hypothesis-generating. The combination of NSE with PRISM III did not significantly improve discrimination over PRISM III alone (DeLong *p* = 0.103), and the absolute AUC gain (+0.058) is modest. PRISM III, which is inexpensive and immediately available at the bedside, remains the appropriate first-line severity assessment in pediatric CSE.
- Prospective multicenter studies with standardized serial NSE sampling, formal long-term neurodevelopmental follow-up, and external validation are required before serum NSE can be advocated for routine clinical use as a prognostic biomarker in pediatric status epilepticus.

**Abstract:**

Background/Objectives: Serum neuron-specific enolase (NSE) is a biomarker of neuronal injury, but its prognostic role in pediatric convulsive status epilepticus (CSE) remains uncertain. We evaluated the association between serum NSE levels and short-term neurological outcome, assessed model calibration with internal bootstrap validation, and examined whether NSE provides incremental discrimination beyond established clinical severity scores. Methods: This was a single-center retrospective cohort study of children aged 1 month to 18 years admitted to a tertiary pediatric intensive care unit (PICU) with CSE as the primary admission diagnosis between January 2024 and November 2025. The primary outcome was poor neurological outcome at hospital discharge, defined as a worsening of ≥1 point in the Pediatric Cerebral Performance Category (PCPC) score from baseline (ΔPCPC ≥ 1) or in-hospital death. A multivariable logistic regression model adjusting for NSE, PRISM III, acute symptomatic etiology, and mechanical ventilation was developed, with bootstrap optimism-corrected internal validation (2000 resamples) and formal calibration assessment. Separate models for in-hospital mortality and for neurological deterioration among survivors were conducted as secondary analyses. Diagnostic operating characteristics were reported with 95% Wilson confidence intervals. The study followed the STROBE and TRIPOD reporting guidelines. Results: Of 132 children included (median age 26 months, 56.1% male), 60 (45.5%) had a poor neurological outcome including 18 deaths (13.6%). Serum NSE was significantly higher in the poor-outcome group (median 22.0 vs. 14.4 μg/L; *p* < 0.001). In the primary multivariable model, NSE (adjusted OR 1.11 per μg/L; 95% CI 1.06–1.19; *p* = 0.001) and PRISM III (adjusted OR 1.15; 95% CI 1.03–1.37; *p* = 0.013) were independently associated with poor outcome. The model showed acceptable calibration (Hosmer–Lemeshow *p* = 0.130) and a bootstrap optimism-corrected AUC of 0.759. NSE remained independently associated with both in-hospital mortality (aOR 1.13) and with ΔPCPC ≥ 1 in survivors (aOR 1.09). The AUC for NSE alone was 0.741 (95% CI 0.65–0.82) for poor outcome and 0.885 (0.79–0.96) for mortality. The combined PRISM III + NSE model showed a numerically higher but not statistically significant AUC compared with PRISM III alone (0.784 vs. 0.726; DeLong *p* = 0.103). Conclusions: Higher serum NSE is independently associated with adverse short-term neurological outcome and mortality in pediatric CSE, including in survivor-only analysis. However, the present data do not demonstrate clinically meaningful incremental prognostic value beyond PRISM III, and the proposed cutoff was derived and tested in the same cohort and is therefore optimistic. These findings are hypothesis-generating and require external validation in prospective multicenter cohorts with serial sampling and long-term neurodevelopmental follow-up before routine clinical use can be advocated.

## 1. Introduction

Convulsive status epilepticus (CSE) is one of the most common life-threatening neurological emergencies in childhood, with an estimated annual incidence of 17 to 23 per 100,000 children and the highest occurrence in the first five years of life [1,2]. According to the ILAE framework, status epilepticus is a neurological condition in which the mechanisms responsible for ending seizures fail or mechanisms promoting persistent seizure activity become activated, resulting in prolonged epileptic activity [1]. Despite advances in management, pediatric CSE continues to be associated with significant morbidity, with neurological sequelae developing in up to 22% of survivors and mortality rates reaching up to 20% in intensive care populations [2,3]. Long-term follow-up cohorts show that adverse neurodevelopmental consequences persist for years, with approximately one in four children exhibiting cognitive, behavioral, or motor impairment at eight years after the index CSE episode [4].

Given the potential for seizure-induced brain injury, there is a growing need for biomarkers that can quantify acute neuronal damage and aid in early prognostic stratification. Several neuro-glial injury biomarkers, including neuron-specific enolase (NSE), S100B, glial fibrillary acidic protein, neurofilament light chain, and tau protein, have been investigated in status epilepticus [5,6]. Among these, NSE is the most extensively studied marker of neuronal injury. NSE is an intracellular glycolytic enzyme predominantly found in neurons and neuroendocrine tissues. Following neuronal injury, it may be released into the extracellular compartment and subsequently detected in peripheral blood. Elevated serum NSE levels reflect both neuronal damage and increased blood–brain barrier permeability and have established prognostic value in conditions such as post-anoxic encephalopathy and traumatic brain injury [7,8].

In adult status epilepticus, DeGiorgio and colleagues demonstrated that serum NSE is elevated across all major SE subtypes and correlates with seizure duration and clinical outcome [9,10]. More recently, Hanin et al. reported that serum NSE levels above 17 ng/mL (equivalent to 17 μg/L; 1 ng/mL = 1 μg/L for NSE) were associated with seizure recurrence in patients with refractory SE, and statistical models incorporating NSE predicted outcomes better than existing clinical scores [11,12]. However, evidence in the pediatric population remains limited and inconsistent. A meta-analysis by Mu et al., including 26 studies and 1360 children with epilepsy, confirmed significantly elevated CSF and serum NSE levels in epileptic children compared with controls [13]. Wang et al. found that serum NSE was markedly elevated on the first day of CSE in 57 children and was independently associated with CSE occurrence [14]. Ansari et al. demonstrated a positive correlation between serum NSE levels and the degree of EEG abnormalities in 100 children with CSE [15]. Conversely, Wong et al. reported that seizure-related NSE elevation in children was rare and occurred primarily in those with symptomatic etiologies [16].

Recent evidence syntheses have emphasized that available pediatric studies are limited by small sample sizes, heterogeneous methodologies, and inconsistent timing of biomarker assessment, leaving the prognostic significance of NSE insufficiently clarified [5]. The relationship between NSE levels and functional neurological outcomes using validated scales has not been adequately characterized in pediatric CSE. In addition, it remains unclear whether NSE provides incremental prognostic value beyond established clinical severity scores such as the Pediatric Risk of Mortality III (PRISM III) score, which primarily reflects systemic physiological derangement rather than brain-specific injury.

The aim of this study was therefore to evaluate the prognostic role of serum NSE levels in children with CSE admitted to a pediatric intensive care unit. Specifically, we sought to (1) assess the association between NSE levels and neurological outcome as measured by the Pediatric Cerebral Performance Category (PCPC) scale, (2) determine an exploratory cutoff value for NSE with formal internal validation, and (3) evaluate whether NSE provides additional predictive value beyond PRISM III. The study was designed and reported in accordance with the STROBE guideline for observational studies [17] and the TRIPOD guideline for prediction-model development [18].

## 2. Materials and Methods

### 2.1. Study Design and Ethics Approval

This was a single-center, retrospective, observational cohort study conducted at the Pediatric Intensive Care Unit (PICU) of Gaziantep City Hospital, a tertiary referral center in southeastern Turkey. Medical records of all children admitted to the PICU with a diagnosis of convulsive status epilepticus (CSE) between January 2024 and November 2025 were retrospectively reviewed. The study protocol was approved by the Gaziantep City Hospital Clinical Research Ethics Committee (Approval No: 389/2025; Date: 25 December 2025). Due to the retrospective design, the requirement for individual informed consent was waived by the ethics committee. All procedures were conducted in accordance with the ethical standards of the Declaration of Helsinki.

### 2.2. Study Population

Eligible patients included children aged 1 month to 18 years admitted to the PICU with clinically diagnosed CSE. In accordance with the current ILAE definition, CSE was defined as continuous convulsive seizure activity lasting 5 min or longer, or two or more discrete seizures between which there was incomplete recovery of consciousness [1].

Inclusion criteria were as follows: (1) age between 1 month and 18 years; (2) clinical diagnosis of CSE at the time of PICU admission, with CSE constituting the primary indication for PICU admission; (3) serum NSE measurement obtained within the first 48 h of PICU admission; and (4) documented Pediatric Cerebral Performance Category (PCPC) score assessment both at baseline (pre-admission) and at hospital discharge.

Exclusion criteria included: (1) hemolyzed blood samples, as hemolysis is a well-recognized source of artifactual NSE elevation due to release of non-neuronal enolase from erythrocytes and platelets [7]; (2) known diagnosis of neuroblastoma or other malignancies associated with elevated NSE; (3) SE occurring in the context of cardiac arrest or post-anoxic encephalopathy; (4) traumatic brain injury, as trauma itself causes significant NSE elevation independent of seizure activity [7]; (5) patients with incomplete data precluding primary outcome assessment; and (6) patients who developed status epilepticus as an in-PICU complication of an unrelated primary admitting diagnosis (e.g., post-operative seizures, sepsis-associated seizures arising during PICU stay). In cases of multiple admissions for the same patient during the study period, only the first episode was included in the analysis.

### 2.3. Clinical Definitions and Classification

Patients were categorized into three groups based on their response to antiseizure treatment. Cases in which seizure activity was controlled with first- and second-line antiseizure medications were classified as SE. Refractory status epilepticus (RSE) was defined as persistence of clinical seizure activity despite the administration of at least two appropriately dosed antiseizure medications, including one benzodiazepine and one second-line agent. Super-refractory status epilepticus (SRSE) was defined as seizure activity persisting for 24 h or more after the initiation of continuous anesthetic infusion therapy, or recurrence of seizures upon withdrawal of anesthetic treatment [1,19]. This three-tier severity classification was treated as a baseline clinical characteristic reflecting disease severity at presentation, not as a treatment outcome.

Etiological classification was performed based on the framework proposed by the ILAE Commission on Epidemiology [20] and was applied using available clinical findings, laboratory results, and neuroimaging data. Etiologies were categorized as follows: (1) febrile SE, (2) acute symptomatic—central nervous system (CNS) infection, (3) acute symptomatic—metabolic (e.g., inborn errors of metabolism, hypoglycemia, electrolyte disturbances), (4) acute symptomatic—other causes, (5) remote symptomatic/structural, and (6) unknown/cryptogenic. For multivariable analyses, etiologies were dichotomized into acute symptomatic (CNS infection, metabolic, and other acute causes; *n* = 45) versus other (febrile, remote symptomatic, and unknown/cryptogenic; *n* = 87) on a priori clinical grounds, because acute symptomatic etiologies are recognized to confer the greatest risk of unfavorable outcome in pediatric SE.

### 2.4. Data Collection

Data were retrospectively extracted from patient medical records and the institutional electronic health record system using a standardized data collection form. The following variables were recorded:

- Demographic data: age (months), sex, body weight, and nationality.
- SE characteristics: etiology, seizure duration (minutes), SE severity classification (SE, RSE, or SRSE), number and type of antiseizure medications administered, and first- and second-line treatments used.
- Laboratory data: Serum NSE (μg/L) was the primary biomarker of interest. At our institution, serum NSE measurement is routinely performed as part of the standard neuronal injury assessment panel for all children admitted to the PICU with neurological emergencies, including status epilepticus, encephalitis, traumatic brain injury, and hypoxic–ischemic encephalopathy. For this study, the first available NSE value obtained within 48 h of PICU admission was used. To account for potential variability in sampling time, the interval between SE onset and blood sampling was recorded and considered in subsequent analyses. NSE was measured using an electrochemiluminescence immunoassay (ECLIA) on a Cobas e 411 analyzer (Roche Diagnostics, Mannheim, Germany) (institutional upper reference limit: 25.0 μg/L). Additional laboratory parameters recorded included C-reactive protein (CRP), serum lactate, albumin, and white blood cell count. S100B measurements were available in only 2 patients and were therefore not analyzed further.
- Clinical severity scores: The Pediatric Risk of Mortality III (PRISM III) score was calculated using the worst physiological values recorded within the first 24 h of PICU admission, as originally described by Pollack et al. [21]. The Pediatric Logistic Organ Dysfunction-2 (PELOD-2) score was calculated to quantify the degree of organ dysfunction [22]. The Glasgow Coma Scale (GCS) score was recorded at both admission and discharge [23].
- PICU data: requirement for and duration of invasive mechanical ventilation (MV), need for continuous anesthetic infusion (third-line therapy), PICU length of stay (LOS), and total hospital LOS.
- Neuroimaging and electroencephalography: EEG and MRI findings were not included in the primary analyses because their acquisition was not standardized across the study cohort. Continuous EEG monitoring was unavailable, and routine EEG recordings could only be performed in clinically stable patients. Likewise, MRI examinations were obtained only when patients were clinically stable and the procedure was considered feasible. As a result, both the availability and timing of EEG and MRI assessments varied considerably among patients, introducing potential selection and timing biases. Therefore, these data were collected and reported descriptively but were not incorporated into the regression models. EEG findings were categorized as normal, generalized slowing, focal slowing, epileptiform discharges (focal or generalized), burst-suppression, or electrocerebral inactivity. MRI findings were categorized as normal, chronic structural lesion, nonspecific changes, acute inflammatory changes, hypoxic–ischemic injury, temporal lobe involvement (atrophy or signal change), focal signal change, multifocal lesion, or diffuse edema. The proportions of patients with available EEG and MRI and the categorical breakdown of findings are reported in the Results.

### 2.5. Outcome Assessment

The primary outcome was poor neurological outcome at hospital discharge, assessed using the Pediatric Cerebral Performance Category (PCPC) scale [24]. The PCPC is a six-point ordinal scale that categorizes overall cognitive and functional neurological status: 1 = normal, 2 = mild disability, 3 = moderate disability, 4 = severe disability, 5 = coma or vegetative state, and 6 = brain death or death. Both the baseline (pre-admission) and discharge PCPC scores were assigned retrospectively from the institutional electronic medical record by two independent outcome assessors, a pediatric neurologist (M.Y.) and a pediatric intensivist (I.B.), using standardized PCPC criteria. Outcome assessors were blinded to serum NSE values when assigning PCPC scores; PCPC scoring was based solely on documented clinical functional status. Any discrepancies in score assignment were resolved through consensus discussion, and the final consensus PCPC score was used for all analyses. Formal inter-rater reliability was not assessed and could not be reconstructed retrospectively.

Poor neurological outcome was defined as a worsening of at least one point in the PCPC score from baseline to discharge (ΔPCPC ≥ 1) or in-hospital death. Good outcome was defined as a stable or improved PCPC score at discharge. This change-based definition was selected to account for pre-existing neurological disability, thereby avoiding misclassification of patients with prior impairment as having a poor outcome attributable to SE [24].

Secondary outcomes included: (1) in-hospital mortality, (2) requirement for invasive mechanical ventilation, (3) PICU length of stay, and (4) need for continuous anesthetic infusion therapy (third-line treatment).

### 2.6. Artificial Intelligence (AI) Disclosure

The authors used ChatGPT (OpenAI) for language editing, minor translation support during manuscript preparation, and assistance in the design of figures. No AI tool was used for study design, data analysis, interpretation of findings, or reference selection. All AI-assisted output was critically reviewed and revised by the authors, who take full responsibility for the final content of the manuscript.

### 2.7. Statistical Analysis

Statistical analyses were performed using SPSS for Windows, version 26.0 (IBM Corp., Armonk, NY, USA), MedCalc Statistical Software version 22.0 (MedCalc Software Ltd., Ostend, Belgium), and Python 3.12 with the statsmodels, scikit-learn, and lifelines libraries. The normality of continuous variables was assessed using the Shapiro–Wilk test. Normally distributed variables were expressed as mean ± standard deviation, while non-normally distributed variables were presented as median and interquartile range (IQR). Categorical variables were reported as frequencies and percentages.

Comparisons between the good-outcome and poor-outcome groups were performed using the independent samples *t*-test or Mann–Whitney U test for continuous variables, as appropriate. Categorical variables were compared using the chi-square test or Fisher’s exact test. Correlations between serum NSE levels and continuous clinical variables (seizure duration, PRISM III score, PELOD-2 score, and PICU LOS) were evaluated using Spearman’s rank correlation coefficient.

Multivariable analysis strategy. A tiered multivariable approach was used, respecting the rule of approximately 10 events per predictor [25]. Three prespecified models were fitted: (i) a primary parsimonious model with four predictors (serum NSE, PRISM III score, acute symptomatic etiology [binary], and mechanical ventilation requirement), selected on a priori clinical grounds, with PRISM III serving as the global severity summary to avoid collinearity with overlapping severity proxies (PELOD-2, lactate, RSE/SRSE classification); (ii) a full sensitivity model additionally incorporating RSE/SRSE classification, serum lactate, and NSE sampling time to assess robustness; and (iii) two outcome-stratified models—a mortality-only model (*n* = 132, events = 18) including NSE, PRISM III, and acute symptomatic etiology, and a survivors-only model for neurological deterioration (*n* = 114 after exclusion of deaths, events = 42) including NSE, PRISM III, acute symptomatic etiology, and mechanical ventilation. Variance inflation factors (VIFs) were computed; a threshold of >5 was used to flag collinearity.

Internal validation and calibration. Bootstrap internal validation was conducted with 2000 resamples following the procedure of Steyerberg, Harrell, and colleagues [26], yielding optimism-corrected estimates of the area under the receiver operating characteristic curve (AUC), the calibration slope (which captures multiplicative miscalibration of predicted risk) and the calibration-in-the-large intercept (which captures additive miscalibration of average predicted risk relative to average observed risk). Adjusted odds ratios (aORs) are reported with 95% percentile bootstrap confidence intervals. Model calibration was further assessed by the Hosmer–Lemeshow goodness-of-fit test (10 deciles of predicted risk) [27], the Brier score, and a nonparametric (LOWESS) calibration plot following the recommendations of Van Calster et al. [28].

ROC analysis. The discriminatory performance of serum NSE was evaluated using receiver operating characteristic (ROC) curve analysis. Receiver operating characteristic analysis was used to identify the threshold that provided the best balance between sensitivity and specificity, as determined by the maximum Youden index value. AUC with 95% bootstrap confidence intervals, sensitivity, specificity, positive predictive value (PPV), negative predictive value (NPV), and likelihood ratios at the optimal cutoff were calculated. Diagnostic operating characteristics are reported with 95% Wilson confidence intervals. To evaluate whether NSE provides incremental predictive value beyond established clinical severity scoring, PRISM III alone, NSE alone, and the combined PRISM III + NSE model were compared; AUCs were compared using the nonparametric method of DeLong et al. [29].

Sampling-time analyses. To address potential confounding by NSE sampling time variability, sampling time was entered as a covariate in the full sensitivity model, sampling-time distributions were compared across SE severity classes by the Kruskal–Wallis test, and a prespecified sensitivity analysis restricted the cohort to patients with NSE measured within 24 h of SE onset.

Survival analysis. Kaplan–Meier survival curves were constructed and compared between groups stratified by the optimal NSE cutoff value using the log-rank test, with 95% confidence interval bands and a number-at-risk table.

Missing data. Variables included in primary and sensitivity multivariable models (NSE, PRISM III, acute symptomatic etiology, mechanical ventilation, RSE/SRSE classification, lactate, NSE sampling time) had no missing values; complete-case analysis was therefore equivalent to the full cohort for these models. S100B measurements were available in only 2 patients; therefore, S100B was not analyzed and was not included in multivariable models.

All statistical tests were two-sided, and a *p*-value < 0.05 was considered statistically significant. The reporting of the prediction-model component followed the TRIPOD guideline [18]; STROBE compliance is documented in Supplementary Material S1.

### 2.8. Sample Size Considerations

This study included all consecutive patients meeting the inclusion criteria during the study period. Based on the widely accepted rule of a minimum of 10 events per predictor variable in logistic regression analysis [25], a minimum of 30 to 40 events (poor neurological outcomes) was required to support a model containing 3 to 4 independent variables. The final cohort of 132 patients, including 60 with poor neurological outcome as defined by ΔPCPC ≥ 1 or death, was considered adequate for the planned primary multivariable analysis. The mortality-only model (18 events) is acknowledged as exploratory and was limited to three predictors.

## 3. Results

### 3.1. Patient Characteristics

During the study period, 158 children were admitted to the PICU with convulsive status epilepticus. Of these, 26 were excluded: 11 due to the absence of serum NSE measurement within 48 h, 7 due to traumatic brain injury, 4 due to cardiac arrest-related SE, 3 due to hemolyzed samples, and 1 due to neuroblastoma. The final cohort comprised 132 patients. The median age was 26 months (IQR: 16–53), and 74 patients (56.1%) were male. The overall in-hospital mortality was 13.6% (18/132).

Regarding SE severity, 69 patients (52.3%) were classified as SE, 47 (35.6%) as RSE, and 16 (12.1%) as SRSE. The most common etiology was febrile SE (*n* = 42, 31.8%), followed by unknown/cryptogenic (*n* = 27, 20.5%), acute symptomatic—CNS infection (*n* = 23, 17.4%), remote symptomatic/structural (*n* = 18, 13.6%), acute symptomatic—other (*n* = 14, 10.6%), and acute symptomatic—metabolic (*n* = 8, 6.1%). When dichotomized for multivariable analysis, 45 patients (34.1%) had an acute symptomatic etiology. Mechanical ventilation was required in 44 patients (33.3%). The median PRISM III score was 8 (IQR: 5–10), the median PELOD-2 score was 3 (IQR: 2–6), and the median admission GCS was 9 (IQR: 7–11).

EEG was performed in 105 patients (105/132, 79.5%) and cranial MRI in 92 patients (92/132, 69.7%); as detailed in Section 2.4, the acquisition of both modalities was not standardized across the cohort and findings are reported descriptively. Among patients with available EEG, findings were abnormal in 84 (80.0%); the most frequent abnormal patterns were mild generalized slowing (26 patients, 24.8%), focal slowing (20, 19.0%), and epileptiform discharges (16, 15.2%), with burst-suppression observed in 12 patients (11.4%) and other non-specific findings in 10 (9.5%). Among patients with available MRI, findings were abnormal in 58 (63.0%); chronic structural lesions (16 patients, 17.4%) and nonspecific changes (16, 17.4%) were most common, with hypoxic–ischemic injury observed in 7 patients (7.6%), acute inflammatory changes in 6 (6.5%), temporal lobe involvement in 5 (5.4%), focal signal change in 4 (4.3%), multifocal lesions in 2 (2.2%), and diffuse edema in 2 (2.2%).

### 3.2. Primary Outcome: Neurological Outcome at Discharge

Based on the primary outcome definition (ΔPCPC ≥ 1 from baseline or in-hospital death), 60 patients (45.5%) had a poor neurological outcome, whereas 72 (54.5%) had a good outcome. Among the 60 patients with poor outcome, 18 (30.0%) died and 42 (70.0%) survived with neurological deterioration at discharge.

Serum NSE levels were significantly higher in the poor-outcome group than in the good-outcome group (median 22.0 μg/L [IQR 14.6–32.6] vs. 14.4 μg/L [IQR 11.5–19.3]; *p* < 0.001). The poor-outcome group also had higher PRISM III scores (9.0 [7.0–14.0] vs. 6.0 [4.0–8.0]; *p* < 0.001), higher PELOD-2 scores (4.5 [3.0–8.0] vs. 3.0 [1.0–4.0]; *p* < 0.001), lower admission GCS (8.0 [6.0–9.0] vs. 11.0 [9.0–12.0]; *p* < 0.001), longer seizure duration (35.5 [20.8–59.2] vs. 19.5 [12.0–31.2] minutes; *p* < 0.001), higher serum lactate (2.8 [1.9–4.1] vs. 1.9 [1.1–2.7] mmol/L; *p* < 0.001), and longer PICU stays (6.0 [4.0–9.0] vs. 4.0 [3.0–6.0] days; *p* < 0.001). Mechanical ventilation was more frequent in the poor-outcome group (46.7% vs. 22.2%; *p* = 0.005), as was the proportion of patients with RSE/SRSE classification (61.7% vs. 36.1%; *p* = 0.006). Age (*p* = 0.252), CRP (*p* = 0.127), and NSE sampling time (*p* = 0.928) did not differ significantly between groups. Etiological distribution differed between groups (*p* = 0.013), with CNS infection more frequently observed in the poor-outcome group (28.3% vs. 8.3%). The complete comparison is presented in Table 1.

### 3.3. Correlation Between NSE and Clinical Variables

Serum NSE showed significant positive correlations with seizure duration (Spearman r = 0.325; *p* < 0.001), PRISM III score (r = 0.330; *p* < 0.001), PELOD-2 score (r = 0.281; *p* = 0.001), and PICU length of stay (r = 0.324; *p* < 0.001).

### 3.4. Univariate and Multivariable Logistic Regression

In univariate logistic regression analysis, serum NSE (OR 1.137 per 1 μg/L increase; 95% CI 1.092–1.199; *p* < 0.001), PRISM III (OR 1.231; 95% CI 1.130–1.389; *p* < 0.001), PELOD-2 (OR 1.285; 95% CI 1.157–1.528; *p* < 0.001), seizure duration (OR 1.021 per minute; 95% CI 1.006–1.055; *p* = 0.002), admission GCS (OR 0.609 per point; 95% CI 0.484–0.715; *p* < 0.001), serum lactate (OR 1.819 per mmol/L; 95% CI 1.431–2.524; *p* < 0.001), mechanical ventilation (OR 3.067; 95% CI 1.522–7.154; *p* = 0.004), RSE/SRSE classification (OR 2.846; 95% CI 1.438–6.400; *p* = 0.001), and acute symptomatic etiology (OR 2.853; 95% CI 1.367–6.041; *p* = 0.005) were all significantly associated with poor neurological outcome. CRP (OR 1.013 per mg/L; 95% CI 1.004–1.025; *p* = 0.004) was also significantly associated in univariate analysis (Table 2).

In the primary parsimonious multivariable model, after adjustment for PRISM III, acute symptomatic etiology, and mechanical ventilation, serum NSE remained independently associated with poor neurological outcome (aOR 1.109 per 1 μg/L; 95% CI 1.058–1.190; *p* = 0.001), as did PRISM III (aOR 1.154 per point; 95% CI 1.032–1.368; *p* = 0.013). Acute symptomatic etiology showed an effect in the expected direction but was not significant after adjustment (aOR 1.89; 95% CI 0.82–4.65; *p* = 0.158), and mechanical ventilation was attenuated to non-significance (aOR 1.10; 95% CI 0.39–2.89; *p* = 0.839). The full sensitivity model—additionally incorporating RSE/SRSE classification, serum lactate, and NSE sampling time to test robustness—yielded a consistent NSE effect (aOR 1.094 per μg/L; 95% CI 1.036–1.183; *p* = 0.005); serum lactate also emerged as an independent predictor (aOR 1.310, 95% CI 1.012–1.749; *p* = 0.041), whereas RSE/SRSE classification and NSE sampling time were not associated with outcome. Maximum variance inflation factor was 1.41 in the primary model and 1.72 in the full sensitivity model, indicating no problematic collinearity. Both models are presented side-by-side in Table 3.

### 3.5. Model Performance, Internal Validation, and Separate Outcome Models

**Figure 1:**
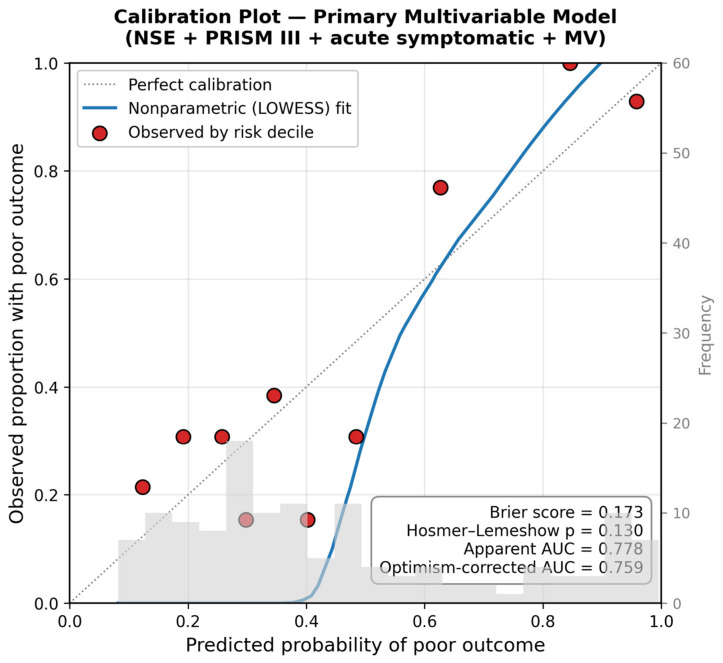
Calibration plot of the primary multivariable model (NSE + PRISM III + acute symptomatic etiology + mechanical ventilation). Red circles indicate observed proportions of poor outcome by decile of predicted risk; the blue line is a LOWESS-smoothed nonparametric calibration curve; the dotted line indicates perfect calibration. The gray histogram shows the distribution of predicted probabilities. Hosmer–Lemeshow *p* = 0.130; Brier score = 0.173; apparent AUC = 0.778; optimism-corrected AUC = 0.759.

Calibration of the primary model. The Hosmer–Lemeshow goodness-of-fit test showed no evidence of poor fit (χ^2^(8) = 12.52; *p* = 0.130). The Brier score was 0.173, and the LOWESS-smoothed calibration plot (Figure 1) demonstrated acceptable agreement between predicted and observed risks across the spectrum of predicted probabilities, with somewhat greater variability in the lower-risk deciles where event counts per decile were small.

Internal validation. Bootstrap internal validation with 2000 resamples yielded an optimism estimate of 0.019 for the AUC. The optimism-corrected calibration slope was 0.900, indicating modest overfitting consistent with the apparent AUC shrinkage; the optimism-corrected calibration-in-the-large intercept was 0.003 (95% bootstrap CI −0.41 to 0.44), with a CI that comfortably brackets zero and indicates no systematic miscalibration of average predicted risk relative to observed risk. This modest optimism is consistent with limited overfitting in the primary parsimonious model but does not substitute for external validation. The full sensitivity model showed a larger optimism-corrected calibration slope shrinkage (0.814) and a slightly wider intercept CI (−0.46 to 0.46).

Separate multivariable models stratified by outcome component. To address the heterogeneity of the composite primary endpoint, two prespecified separate multivariable models were fitted (Table 4): a mortality-only model (*n* = 132, events = 18, restricted to three predictors because of the limited event count) and a survivors-only model (*n* = 114 after exclusion of deaths, events = 42; outcome defined as ΔPCPC ≥ 1). In the mortality-only model, both NSE and PRISM III remained independently associated with in-hospital mortality. In the survivors-only model, NSE remained independently associated with ΔPCPC ≥ 1, while PRISM III showed a borderline association. Among the 114 survivors, median NSE was 18.8 μg/L (IQR 12.9–27.9) in those with neurological deterioration vs. 14.5 μg/L (IQR 11.5–19.3) in those with stable PCPC (Mann–Whitney *p* = 0.004). The optimism-corrected AUC was 0.913 for the mortality-only model and 0.676 for the survivors-only model. For the survivors-only model, the optimism-corrected calibration slope was 0.856 and the calibration intercept was 0.003 (95% bootstrap CI −0.42 to 0.46), again with a CI bracketing zero and consistent with no systematic miscalibration.

The consistency of findings across the composite outcome, mortality-only, and survivors-only analyses suggests that the observed NSE–outcome association is not solely driven by mortality.

### 3.6. ROC Analysis, Prognostic Cutoff, and Sampling-Time Sensitivity

ROC analysis for predicting poor neurological outcome yielded an AUC of 0.741 (95% CI 0.650–0.823) for serum NSE alone. The optimal cutoff determined by the Youden index was 25.7 μg/L, demonstrating the expected high-specificity, low-sensitivity profile of a rule-in marker (Table 5). Because the cutoff was derived and tested in the same cohort, the reported metrics—particularly specificity and PPV—are likely to be optimistic in external populations and are presented as exploratory.

**Figure 2:**
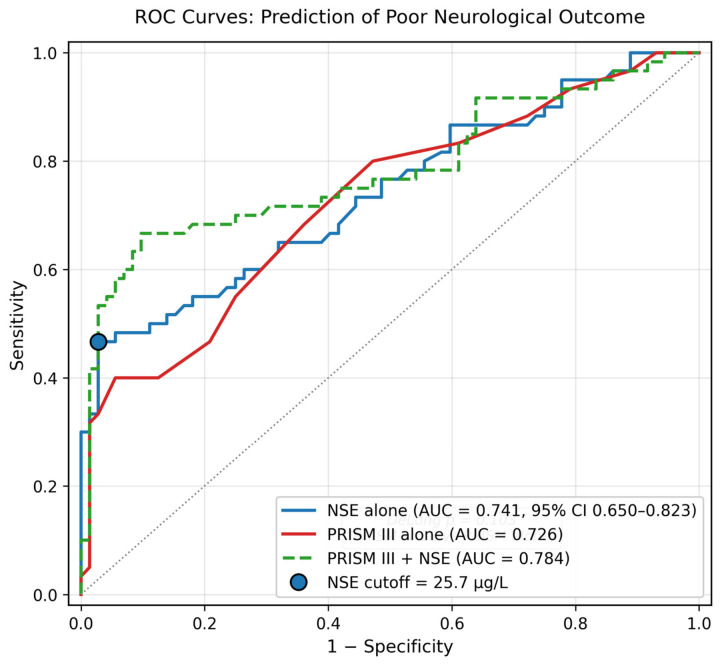
Receiver operating characteristic (ROC) curves for prediction of poor neurological outcome. NSE alone (AUC 0.741, 95% CI 0.650–0.823; blue), PRISM III alone (AUC 0.726; red), and the combined model PRISM III + NSE (AUC 0.784; green dashed). The blue circle indicates the operating point of the exploratory NSE cutoff at 25.7 μg/L. The DeLong test comparing PRISM III + NSE to PRISM III alone was not statistically significant (*p* = 0.103).

PRISM III alone yielded an AUC of 0.726, and PELOD-2 alone an AUC of 0.709. The combined PRISM III + NSE model yielded an AUC of 0.784, a numerical difference of +0.058 over PRISM III alone that did not reach statistical significance (DeLong *p* = 0.103; Table 5, Figure 2.

**Figure 3:**
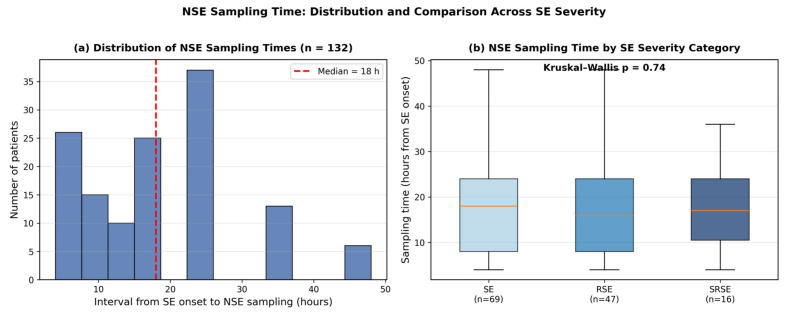
Distribution of NSE sampling times. (**a**) Overall histogram of the interval (hours) between SE onset and serum NSE sampling in the full cohort (*n* = 132); the red dashed line indicates the median (17 h). (**b**) Box plots of sampling time stratified by SE severity category (SE, RSE, SRSE); Kruskal–Wallis *p* = 0.74, indicating no significant difference across severity groups. The orange horizontal line within each box indicates the median value.

NSE sampling-time analyses. Overall, NSE was sampled at a median of 17 h (IQR 8–24) after SE onset. The distribution of sampling intervals was 38.6% ≤ 12 h, 47.0% > 12–24 h, and 14.4% > 24–48 h (Figure 3a). Sampling time did not differ significantly across SE severity categories (SE median 18 h, RSE 16 h, SRSE 17 h; Kruskal–Wallis *p* = 0.74; Figure 3b). NSE sampling time was not significantly associated with poor outcome (Mann–Whitney *p* = 0.928), and when entered as a covariate in the full sensitivity multivariable model (Table 3, right column) it did not materially change the NSE effect estimate. A prespecified sensitivity analysis restricted to 113 patients (85.6%) with NSE measured within 24 h of SE onset yielded an AUC of 0.763, consistent with the overall analysis.

### 3.7. Secondary Outcomes

**Figure 4:**
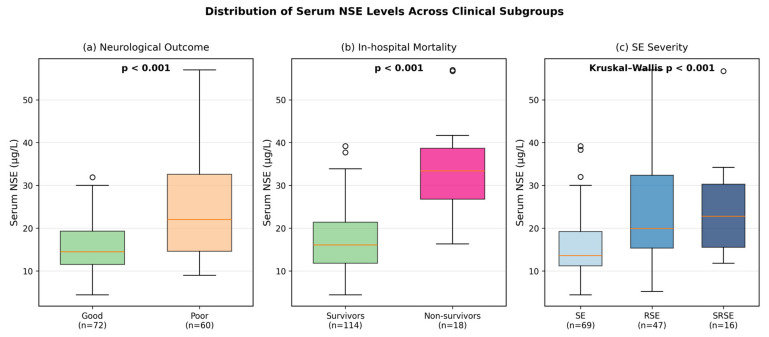
Distribution of serum NSE levels across clinical subgroups, expressed as box plots (median, IQR, and whiskers to 1.5 × IQR). Circles indicate outlier values beyond 1.5 × IQR. (**a**) Good vs. poor neurological outcome (*p* < 0.001); (**b**) survivors vs. non-survivors (*p* < 0.001); (**c**) SE severity categories—SE, RSE, SRSE (Kruskal–Wallis *p* < 0.001). The orange horizontal line within each box indicates the median value.

SE severity. NSE levels increased across SE severity categories: SE (median 13.6 μg/L [IQR 11.2–19.2]), RSE (19.9 [15.3–32.4]), and SRSE (22.8 [15.5–30.3]; Kruskal–Wallis *p* < 0.001). The distributions of NSE across outcome groups, mortality status, and severity categories are shown in Figure 4.

Mechanical ventilation. Patients requiring mechanical ventilation had higher NSE levels (median 20.5 μg/L [IQR 17.2–32.9] vs. 14.4 μg/L [IQR 11.6–21.4]; *p* < 0.001).

**Figure 5:**
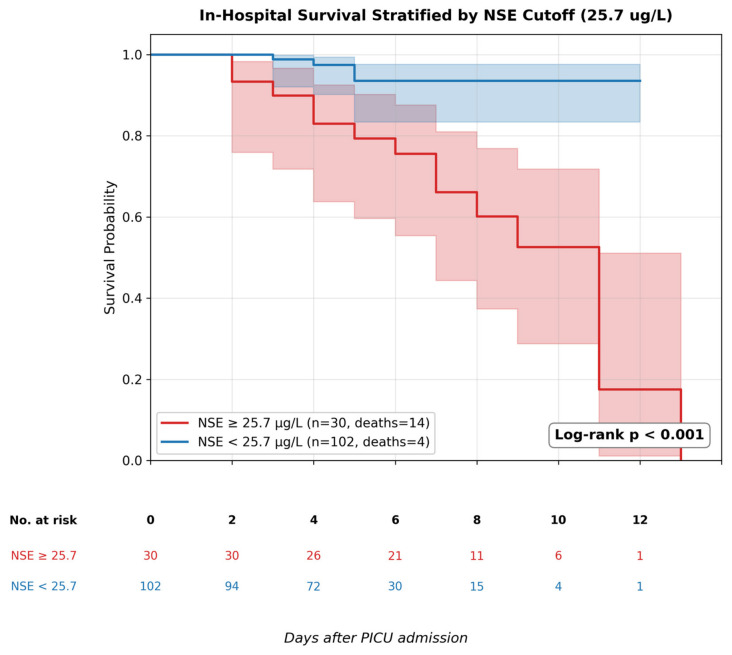
Kaplan–Meier in-hospital survival curves stratified by NSE cutoff (25.7 μg/L). Log-rank *p* < 0.001. Shaded bands indicate 95% confidence intervals; the table below the *x*-axis shows the number at risk at 2-day intervals.

Mortality. Serum NSE was significantly higher in non-survivors compared with survivors (median 33.4 μg/L [IQR 26.8–38.7] vs. 16.1 [IQR 11.8–21.4]; *p* < 0.001). The AUC for NSE in predicting in-hospital mortality was 0.885 (95% CI 0.79–0.96). Kaplan–Meier analysis with 95% confidence interval bands and a number-at-risk table demonstrated significantly lower survival probability in patients with NSE ≥ 25.7 μg/L (log-rank *p* < 0.001; 14/30 deaths above the cutoff vs. 4/102 below; Figure 5).

### 3.8. Subgroup Analyses by Etiology and Imaging Findings

Patients with acute symptomatic etiologies (*n* = 45) had higher median NSE levels compared with those with other etiologies (*n* = 87): 19.3 μg/L (IQR 12.7–32.1) vs. 16.4 μg/L (IQR 11.4–21.2); *p* = 0.019. Poor outcomes were also more frequent in the acute symptomatic group (62.2% vs. 36.8%).

In exploratory subgroup analyses restricted to patients with available imaging, NSE levels did not differ significantly between patients with abnormal vs. normal EEG (median 18.2 vs. 14.9 μg/L; *p* = 0.12; *n* = 105) or between patients with abnormal vs. normal MRI (median 18.7 vs. 15.8 μg/L; *p* = 0.09; *n* = 92), although the direction of effect was consistent with higher NSE in patients with imaging abnormalities. These subgroup comparisons should be interpreted with caution given the non-standardized acquisition of EEG and MRI across the cohort (Section 2.4).

## 4. Discussion

In this single-center retrospective cohort of 132 children with convulsive status epilepticus admitted to a tertiary PICU, higher serum neuron-specific enolase measured within 48 h of admission was independently associated with poor neurological outcome at hospital discharge (adjusted OR 1.11 per μg/L; 95% CI 1.06–1.19; *p* = 0.001) and showed good discrimination for in-hospital mortality (AUC 0.885; 95% CI 0.79–0.96). The association persisted in separate analyses restricted to mortality (*n* = 132, events = 18) and to neurological deterioration among survivors (*n* = 114, events = 42), arguing against the possibility that the composite-endpoint finding was driven exclusively by deaths. An exploratory cutoff of 25.7 μg/L, closely approximating the institutional upper reference limit (25.0 μg/L), identified high-risk patients with high specificity (97.2%) and PPV (93.3%) but limited sensitivity (46.7%), consistent with a rule-in rather than screening profile. However, the combination of NSE with PRISM III did not significantly improve discrimination over PRISM III alone (ΔAUC +0.058; DeLong *p* = 0.103), and the present data do not demonstrate clinically meaningful incremental prognostic value of NSE beyond a routinely calculated severity score. These findings are hypothesis-generating and require prospective external validation with serial sampling and long-term neurodevelopmental follow-up before serum NSE can be recommended for routine use.

### 4.1. NSE as a Prognostic Biomarker in Pediatric CSE

Several biological considerations support the plausibility of an NSE–outcome association in pediatric SE. NSE is a glycolytic enzyme located in the cytoplasm of neurons and neuroendocrine cells; its release into the extracellular compartment and subsequent diffusion into the systemic circulation reflect disruption of neuronal membrane integrity, which occurs after both reversible and irreversible neuronal injury [7]. Prolonged seizure activity is known to trigger excitotoxic cascades, mitochondrial dysfunction, and selective neuronal death in vulnerable regions such as the hippocampus and limbic cortex [5,6]. The dose–response relationship that we observed across SE → RSE → SRSE (median 13.6 → 19.9 → 22.8 μg/L) and across severity-stratified analyses is consistent with the hypothesis that NSE elevation captures the cumulative burden of seizure-induced neuronal injury rather than a single discrete event.

Our findings extend a literature in which the prognostic value of NSE in pediatric SE has been investigated predominantly in small or heterogeneous cohorts. Wang and colleagues, in 57 children with CSE, observed elevated NSE on the first day of CSE and an independent association with CSE occurrence [14]. Ansari et al. demonstrated a positive correlation between serum NSE and the degree of EEG abnormalities in 100 children with CSE [15]. A meta-analysis of 26 studies by Mu et al. confirmed significantly elevated serum and CSF NSE in epileptic children compared with controls [13]. However, none of these studies systematically evaluated functional neurological outcome using a validated change-based instrument such as PCPC, examined incremental prognostic value over a clinical severity score, or assessed model calibration with internal bootstrap validation. The present study addresses these gaps.

### 4.2. Exploratory NSE Cutoff and Its Operational Profile

The exploratory cutoff of 25.7 μg/L identified by the Youden index in this cohort closely corresponds to the institutional upper reference limit for serum NSE (25.0 μg/L) and would therefore not require any nonstandard interpretive threshold in clinical practice if subsequently externally validated. Its operational profile—sensitivity 46.7%, specificity 97.2%, PPV 93.3%, positive likelihood ratio 16.8—places it firmly in the category of a rule-in test: an NSE measurement above the threshold conveys strong evidence of poor outcome, but a measurement below the threshold does not reliably exclude it. This is consistent with patterns described in adult SE [11,12] and reflects the heterogeneity of SE etiologies, in which severe seizure-induced neuronal injury can occur without massive NSE elevation, and conversely, in which moderate elevation may reflect non-seizure pathology.

It must be emphasized that because the cutoff was derived and tested in the same cohort, the reported metrics are likely to be optimistic in external populations. The expected loss of specificity and PPV on independent validation, even with the same cutoff, has been well documented in the prediction-modeling literature [28]. Bootstrap optimism correction for AUC (0.778 → 0.759) provides one estimate of this overfitting, but external validation in a geographically and demographically distinct cohort remains essential before the cutoff can be advocated for clinical decision-making.

### 4.3. Incremental Value over PRISM III

A central question in any prognostic biomarker study is whether the new marker adds clinically meaningful information beyond what is already obtainable from routinely collected data. In our cohort, the model combining PRISM III and NSE yielded a numerically higher AUC than PRISM III alone (0.784 vs. 0.726), but this difference did not reach statistical significance (DeLong *p* = 0.103) and the absolute improvement of 0.058 is modest. The present data therefore do not establish that NSE adds independent prognostic information beyond a routinely calculated severity score. PRISM III is inexpensive, immediately available at the bedside, requires no laboratory turnaround time, and is validated across diverse pediatric intensive care populations [21]. Given these practical advantages, PRISM III remains the appropriate first-line severity assessment in pediatric CSE, and any future advocacy for NSE as a clinical prognostic marker would need to demonstrate either statistically and clinically meaningful incremental discrimination, improved calibration in important risk strata, or a specific clinical decision threshold (such as triage to advanced neuromonitoring or anesthetic therapy) at which the combined model outperforms the severity score alone. NSE may still capture brain-specific aspects of injury that are not represented in a global physiological severity score, and the survivors-only analysis (Section 3.5) suggests that this signal persists when systemic severity is partially controlled by excluding deaths; however, this hypothesis requires prospective validation in adequately powered multicenter cohorts.

### 4.4. Brain-Specific Injury Versus Systemic Illness Severity

A second and conceptually related question is whether the NSE–outcome association reflects brain-specific neuronal injury or whether it primarily mirrors overall illness severity. Several lines of evidence in our data argue for at least partial brain-specificity. First, NSE retained an independent association with poor outcome after adjustment for PRISM III (aOR 1.11 per μg/L), and after additional adjustment for RSE/SRSE classification, serum lactate, mechanical ventilation, and NSE sampling time (Table 3, full sensitivity column; aOR 1.09 per μg/L). Second, NSE remained significantly associated with neurological deterioration in the survivors-only analysis (*n* = 114, events = 42), in which the most severe form of systemic illness—death—is excluded. Third, the biological rationale is direct: NSE is largely neuron-specific and is released upon membrane disruption following neuronal injury [7].

However, several caveats apply. NSE is also present in neuroendocrine tissues and is released from erythrocytes during hemolysis [7], although hemolyzed samples were excluded from our analysis. Serum NSE concentrations may further be influenced by the integrity of the blood–brain barrier, which is itself altered in systemic critical illness and during prolonged seizure activity [30]. Moreover, the AUC of NSE alone in the present cohort (0.741) is in the moderate range, indicating that NSE cannot be expected to function as a stand-alone diagnostic test. We therefore interpret our findings as supporting NSE as an adjunctive marker that may provide complementary information about neuronal injury, rather than as a replacement for clinical severity assessment.

### 4.5. Etiological Considerations

Acute symptomatic etiologies—particularly CNS infection—were more frequent in patients with poor outcome (28.3% vs. 8.3%), and our subgroup analysis showed higher median NSE in the acute symptomatic group (19.3 vs. 16.4 μg/L; *p* = 0.019). In the primary multivariable model, acute symptomatic etiology showed an effect in the expected direction (aOR 1.89; 95% CI 0.82–4.65) but did not reach statistical significance after adjustment, possibly because PRISM III partially captures the systemic consequences of CNS infection (fever, leukocytosis, acidosis) and because of the modest event count for this subgroup. The persistence of an NSE effect even after adjustment for etiology argues against the simplistic view that NSE elevation in this cohort is merely a surrogate marker of CNS infection. Nonetheless, NSE is not exclusively elevated in seizure-induced injury, and our findings should be interpreted in the context of an etiologically heterogeneous cohort. Future studies stratified by etiology would help clarify whether the prognostic information conveyed by NSE differs across etiological subgroups.

### 4.6. Limitations

This study has several important limitations.

First, the retrospective single-center design limits both generalizability and causal inference. The cohort represents children admitted to a single tertiary referral PICU in southeastern Turkey, in which a substantial proportion of patients are transferred from peripheral centers, potentially affecting both severity and timing of NSE sampling.

Second, the NSE sampling time was variable (median 17 h, range 4–48 h post-SE onset). Although our analyses did not detect a systematic effect of sampling time on outcome and severity-stratified sampling times were similar (Section 3.6 and Figure 2), serial NSE sampling—at admission, 24 h, and 48–72 h—is needed to characterize the temporal trajectory of NSE release and identify the optimal sampling window. Delayed hospital or PICU presentation, particularly in transferred patients, may bias NSE values toward earlier or later positions on the release curve.

Third, the outcome measure (discharge PCPC) captures only short-term functional neurological status and cannot detect delayed cognitive, behavioral, or epilepsy-related sequelae. Baseline and discharge PCPC scores were assigned retrospectively by two independent outcome assessors who were blinded to serum NSE values, and discrepancies were resolved through consensus discussion. However, formal inter-rater reliability statistics (e.g., weighted Cohen’s kappa) were not calculated and could not be reconstructed retrospectively. Therefore, the reproducibility of the retrospective PCPC assessments cannot be formally quantified and should be considered a limitation of the study. Long-term cohort data from Pujar et al. demonstrate that approximately one-quarter of children with CSE exhibit ongoing neurodevelopmental impairment at 8 years of follow-up, with cognitive, motor, and behavioral domains affected beyond what is captured at hospital discharge [4]. Prospective studies with structured neurodevelopmental assessment at 12 and 24 months are required before NSE can be advocated as a long-term prognostic marker.

Fourth, the cutoff of 25.7 μg/L was derived and tested in the same cohort, and despite bootstrap optimism correction the reported metrics—particularly specificity and PPV—should be considered optimistic; external validation in geographically and demographically distinct cohorts is the essential next step before clinical use of any specific threshold, in line with the TRIPOD recommendations [18].

Fifth, patients who developed status epilepticus as an in-PICU complication of an unrelated primary admitting diagnosis (for example, post-operative seizures, sepsis-associated seizures arising during PICU stay) were not included in this cohort. This may limit generalizability of our findings to PICU-acquired status epilepticus, in which baseline NSE values may be affected by the antecedent critical illness.

Finally, we did not perform continuous EEG monitoring or measure complementary biomarkers (CSF NSE, neurofilament light chain, glial fibrillary acidic protein) that may further characterize seizure-induced neuronal injury.

### 4.7. Implications and Future Directions

In summary, the present findings should be interpreted as hypothesis-generating. They suggest that serum NSE measured during the first 48 h of PICU admission contains prognostic information that is independent of the PRISM III score and that persists in survivor-only analysis, but they do not establish that NSE provides clinically meaningful incremental discrimination over a routinely calculated severity score. The exploratory cutoff of 25.7 μg/L identifies a small but high-risk subgroup with a strong rule-in profile, but it requires external validation.

The next steps required to advance this field include: (1) prospective multicenter cohort studies with standardized serial NSE sampling at admission, 24 h, and 48–72 h; (2) structured neurodevelopmental follow-up at 12 and 24 months using validated developmental assessment tools; (3) comparative evaluation of NSE alongside complementary biomarkers (S100B, glial fibrillary acidic protein, neurofilament light chain); (4) etiology-stratified analyses, particularly in children with CNS infection-associated SE; and (5) clinical utility studies addressing whether NSE-guided risk stratification at PICU admission influences clinically meaningful decisions such as the timing of advanced neuromonitoring, anesthetic therapy initiation, or family communication regarding prognosis. Until such evidence is available, NSE should not replace clinical severity scoring in routine practice.

## 5. Conclusions

In this single-center retrospective cohort of 132 children with convulsive status epilepticus, higher serum neuron-specific enolase measured within 48 h of PICU admission was independently associated with poor short-term neurological outcome and with in-hospital mortality, including in a survivors-only analysis restricted to neurological deterioration. An exploratory cutoff of 25.7 μg/L identified high-risk children with high specificity and a strong rule-in profile, but with limited sensitivity. However, the combination of NSE with PRISM III did not significantly improve discrimination over PRISM III alone, and the present data do not demonstrate clinically meaningful incremental prognostic value of NSE beyond a routinely calculated severity score. PRISM III, which is inexpensive and immediately available at the bedside, remains the appropriate first-line severity assessment in pediatric CSE. The cutoff was derived and tested in the same cohort and is therefore optimistic; bootstrap optimism correction yielded a modest reduction in AUC. These findings are hypothesis-generating and require external validation in prospective multicenter cohorts with serial sampling and long-term neurodevelopmental follow-up before serum NSE can be advocated for routine clinical use as a prognostic biomarker in pediatric status epilepticus.

## Figures and Tables

**Table 1:** Comparison of demographic and clinical characteristics between good and poor neurological outcome groups.

Variable	Total (*n* = 132)	Good Outcome (*n* = 72)	Poor Outcome (*n* = 60)	*p*
Demographics				
Male sex, *n* (%)	74 (56.1)	44 (61.1)	30 (50.0)	0.269
Age, months, median (IQR)	26 (16–53)	29 (17–60)	24 (14–42)	0.252
SE Characteristics				
Seizure duration, min, median (IQR)	26 (16–43)	20 (12–31)	36 (21–59)	<0.001
SE, *n* (%)	69 (52.3)	46 (63.9)	23 (38.3)	0.004
RSE, *n* (%)	47 (35.6)	22 (30.6)	25 (41.7)	
SRSE, *n* (%)	16 (12.1)	4 (5.6)	12 (20.0)	
Etiology, *n* (%)				
Febrile SE	42 (31.8)	28 (38.9)	14 (23.3)	0.013
Acute symptomatic—CNS infection	23 (17.4)	6 (8.3)	17 (28.3)	
Acute symptomatic—metabolic	8 (6.1)	2 (2.8)	6 (10.0)	
Acute symptomatic—other	14 (10.6)	9 (12.5)	5 (8.3)	
Remote symptomatic/structural	18 (13.6)	12 (16.7)	6 (10.0)	
Unknown/cryptogenic	27 (20.5)	15 (20.8)	12 (20.0)	
Acute symptomatic (combined), *n* (%)	45 (34.1)	17 (23.6)	28 (46.7)	0.006
Clinical Severity Scores				
PRISM III, median (IQR)	8 (5–10)	6 (4–8)	9 (7–14)	<0.001
PELOD-2, median (IQR)	3 (2–6)	3 (1–4)	4 (3–8)	<0.001
Admission GCS, median (IQR)	9 (7–11)	11 (9–12)	8 (6–9)	<0.001
Laboratory Parameters				
Serum NSE, μg/L, median (IQR)	17.1 (12.0–23.7)	14.4 (11.5–19.3)	22.0 (14.6–32.6)	<0.001
NSE sampling time, h, median (IQR)	18 (8–24)	18 (8–24)	17 (8–24)	0.928
Lactate, mmol/L, median (IQR)	2.3 (1.4–3.3)	1.9 (1.1–2.7)	2.8 (1.9–4.1)	<0.001
CRP, mg/L, median (IQR)	14.0 (7.9–37.3)	13.9 (7.2–27.3)	16.4 (8.2–64.2)	0.127
PICU Data				
Mechanical ventilation, *n* (%)	44 (33.3)	16 (22.2)	28 (46.7)	0.005
PICU LOS, days, median (IQR)	5 (4–7)	4 (3–6)	6 (4–9)	<0.001
In-hospital mortality, *n* (%)	18 (13.6)	0 (0)	18 (30.0)	–

Data are presented as median (IQR) or *n* (%). Poor outcome defined as ΔPCPC ≥ 1 or death. *p*-values by Mann–Whitney U test (continuous) or chi-square test (categorical). SE = Status Epilepticus, RSE = Refractory Status Epilepticus, SRSE = Super-Refractory Status Epilepticus, CNS = Central Nervous System, PRISM III = Pediatric Risk of Mortality III, PELOD-2 = Pediatric Logistic Organ Dysfunction-2, GCS = Glasgow Coma Scale, NSE = Neuron-Specific Enolase, CRP = C-Reactive Protein, PICU = Pediatric Intensive Care Unit, PICU LOS = Pediatric Intensive Care Unit Length of Stay.

**Table 2:** Univariate logistic regression analysis for predictors of poor neurological outcome.

Variable	OR	95% CI	*p*
Serum NSE (per 1 μg/L)	1.137	1.092–1.199	<0.001
PRISM III (per point)	1.231	1.130–1.389	<0.001
PELOD-2 (per point)	1.285	1.157–1.528	<0.001
Age (per month)	0.996	0.985–1.004	0.306
Seizure duration (per min)	1.021	1.006–1.055	0.002
Admission GCS (per point)	0.609	0.484–0.715	<0.001
Serum lactate (per mmol/L)	1.819	1.431–2.524	<0.001
CRP (per mg/L)	1.013	1.004–1.025	0.004
Mechanical ventilation	3.067	1.522–7.154	0.004
RSE/SRSE	2.846	1.438–6.400	0.001
Acute symptomatic etiology	2.853	1.367–6.041	0.005

OR, odds ratio; CI, confidence interval (bootstrap, 2000 resamples).

**Table 3:** Multivariable logistic regression models for the composite primary outcome (ΔPCPC ≥ 1 or in-hospital death; *n* = 132, events = 60). Two models are presented side-by-side: a parsimonious model with four a priori covariates, and a full sensitivity model adding three additional severity proxies.

Predictor	Primary Parsimonious			Full Sensitivity		
	aOR	95% CI	*p*	aOR	95% CI	*p*
Serum NSE (per 1 μg/L)	1.109	1.058–1.190	0.001	1.094	1.036–1.183	0.005
PRISM III (per point)	1.154	1.032–1.368	0.013	1.122	1.001–1.298	0.048
Acute symptomatic etiology	1.889	0.823–4.647	0.158	1.612	0.652–4.012	0.301
Mechanical ventilation	1.102	0.394–2.889	0.839	0.876	0.291–2.654	0.812
RSE/SRSE classification	—	—	—	1.342	0.572–3.157	0.500
Serum lactate (per mmol/L)	—	—	—	1.310	1.012–1.749	0.041
NSE sampling time (per hour)	—	—	—	1.006	0.968–1.055	0.745
Model performance						
Apparent AUC	0.778	—	—	0.794	—	—
Optimism-corrected AUC	0.759	—	—	0.757	—	—
Hosmer–Lemeshow *p*	0.130	—	—	0.176	—	—
Brier score	0.173	—	—	0.171	—	—
Calibration slope (optimism-corrected)	0.900	—	—	0.814	—	—
Calibration intercept (optimism-corrected)	0.003	—	—	−0.005	—	—
Maximum VIF	1.41	—	—	1.72	—	—

aOR, adjusted odds ratio; CI, confidence interval; VIF, variance inflation factor. 95% CIs estimated by 2000-resample percentile bootstrap. Optimism-corrected AUC, calibration slope, and calibration intercept obtained by Harrell’s bootstrap procedure (2000 resamples). Hosmer–Lemeshow goodness-of-fit test computed with 10 deciles of predicted risk. The primary parsimonious model was prespecified with four a priori clinically justified covariates respecting the events-per-variable rule (60 events/4 predictors = 15). The full sensitivity model additionally incorporates RSE/SRSE classification, serum lactate, and NSE sampling time to test robustness against over-parameterization of severity proxies. Dashes (—) indicate predictors not included in that model.

**Table 4:** Outcome-stratified multivariable logistic regression models. The mortality-only model includes all 132 patients with in-hospital mortality as the outcome; the survivors-only model excludes the 18 in-hospital deaths to isolate the association of NSE with neurological deterioration among children who survived to discharge.

Predictor	Mortality-Only			Survivors-Only (ΔPCPC ≥ 1)		
	(*n* = 132, Events = 18)			(*n* = 114, Events = 42)		
	aOR	95% CI	*p*	aOR	95% CI	*p*
Serum NSE (per 1 μg/L)	1.126	1.043–1.322	0.003	1.094	1.035–1.178	0.005
PRISM III (per point)	1.277	1.093–2.010	0.002	1.127	0.991–1.370	0.056
Acute symptomatic etiology	1.708	0.242–21.36	0.469	2.014	0.876–5.047	0.129
Mechanical ventilation	—	—	—	0.740	0.235–1.990	0.559
Model performance						
Apparent AUC	0.927	—	—	0.706	—	—
Optimism-corrected AUC	0.913	—	—	0.676	—	—
Hosmer–Lemeshow *p*	—	—	—	0.243	—	—
Brier score	0.078	—	—	0.215	—	—
Calibration slope (optimism-corrected)	—	—	—	0.856	—	—
Calibration intercept (optimism-corrected)	—	—	—	0.003	—	—

aOR, adjusted odds ratio; CI, confidence interval. 95% CIs estimated by 2000-resample percentile bootstrap. Optimism-corrected AUC, calibration slope, and calibration intercept obtained by Harrell’s bootstrap procedure (2000 resamples). The mortality-only model was restricted to three predictors because of the limited event count (18); Hosmer–Lemeshow test, calibration slope, and calibration intercept not reported because of sparse cells with 18 events distributed across deciles of predicted risk. Dashes (—) indicate predictors not included in that model or metrics not reliably estimable.

**Table 5:** ROC analysis for predicting poor neurological outcome.

Model	AUC	95% CI	Cutoff	Sens %	Spec %	PPV %	NPV %	LR+	LR−
NSE alone	0.741	0.650–0.823	25.7 μg/L	46.7 (34.6–59.1)	97.2 (90.4–99.2)	93.3 (78.7–98.2)	68.6 (59.1–76.8)	16.8	0.55
PRISM III alone	0.726	0.637–0.815	–	–	–	–	–	–	–
PELOD-2 alone	0.709	0.619–0.799	–	–	–	–	–	–	–
PRISM III + NSE	0.784	0.706–0.862	–	–	–	–	–	–	–

ΔAUC (PRISM III + NSE vs. PRISM III alone) = +0.058; DeLong test *p* = 0.103. NSE cutoff determined by the Youden index, derived and tested in the same cohort (exploratory). Diagnostic operating characteristics with 95% Wilson confidence intervals; AUC 95% CIs by 2000-resample bootstrap. Sens = Sensitivity; Spec = Specificity; PPV = Positive Predictive Value; NPV = Negative Predictive Value; LR+ = Positive Likelihood Ratio; LR− = Negative Likelihood Ratio.

## Data Availability

The data presented in this study are available on reasonable request from the corresponding author. The data are not publicly available due to ethical and institutional restrictions related to patient confidentiality.

## Supplementary Materials

The following supporting information can be downloaded at: https://www.mdpi.com/article/10.3390/children13060820/s1, Supplementary Material S1—STROBE checklist for the observational cohort component of this study.

## Abbreviations

The following abbreviations are used in this manuscript:

CSE	Convulsive Status Epilepticus
CI	Confidence Interval
CNS	Central Nervous System
CRP	C-Reactive Protein
EEG	Electroencephalography
GCS	Glasgow Coma Scale
ICU	Intensive Care Unit
IQR	Interquartile Range
LOS	Length of Stay
MRI	Magnetic Resonance Imaging
MV	Mechanical Ventilation
NSE	Neuron-Specific Enolase
OR	Odds Ratio
aOR	Adjusted Odds Ratio
PCPC	Pediatric Cerebral Performance Category
PELOD-2	Pediatric Logistic Organ Dysfunction-2
PICU	Pediatric Intensive Care Unit
PPV	Positive Predictive Value
NPV	Negative Predictive Value
LR+	Positive Likelihood Ratio
LR−	Negative Likelihood Ratio
PRISM III	Pediatric Risk of Mortality III
ROC	Receiver Operating Characteristic
AUC	Area Under the Curve
RSE	Refractory Status Epilepticus
SD	Standard Deviation
SE	Status Epilepticus
SRSE	Super-Refractory Status Epilepticus
STROBE	Strengthening the Reporting of Observational Studies in Epidemiology
TRIPOD	Transparent Reporting of a Multivariable Prediction Model for Individual Prognosis Or Diagnosis
ILAE	International League Against Epilepsy
VIF	Variance Inflation Factor
