# Supplementary material for: Serum Neuron-Specific Enolase as a Prognostic Biomarker in Pediatric Convulsive Status Epilepticus: A Single-Center Retrospective Cohort Study
Source: Children (Basel). 2026 Jun 15;13(6):820. doi: 10.3390/children13060820 (PMC13297551; doi:10.3390/children13060820)
Supplement: Supplementary file 1 [file children-13-00820-s001.zip › children-4307917-supplementary.pdf]

Supplementary Material S1

STROBE Statement — Checklist of items that should be included in reports of cohort studies

Manuscript: Serum Neuron-Specific Enolase as a Prognostic Biomarker in Pediatric Convulsive Status Epilepticus: A Single-Center Retrospective Cohort Study

Journal: Children (MDPI) | Manuscript ID: children-4307917

The following checklist documents compliance of this manuscript with the STROBE (Strengthening the Reporting of Observational Studies in Epidemiology) Statement for cohort studies [von Elm et al., Ann Intern Med. 2007;147(8):573–577]. For each of the 22 STROBE items, the location in the revised manuscript is indicated.

STROBE Section	Item No.	Recommendation	Location in manuscript
Title and abstract			
Title and abstract	**1**	(a) Indicate the study's design with a commonly used term in the title or the abstract.	Title: "...A Single-Center Retrospective Cohort Study" (page 1, lines 2–4). Abstract — Methods section explicitly states "single-center retrospective cohort study" (page 1, Methods sentence).
Title and abstract	**1**	(b) Provide in the abstract an informative and balanced summary of what was done and what was found.	Abstract is structured (Background/Objectives, Methods, Results, Conclusions; page 1). Conclusions softened to hypothesis-generating framing in the revised version ("the present data do not demonstrate clinically meaningful incremental prognostic value").
Introduction			
Introduction	**2**	Background/rationale: Explain the scientific background and rationale for the investigation being reported.	Section 1 (Introduction). Five paragraphs covering: epidemiology and burden of pediatric CSE [refs 1–3]; biological role of NSE and other neuro-glial markers [refs 5–8]; existing adult [refs 9–12] and pediatric [refs 13–16] evidence; identified gaps in the pediatric literature [ref 5].
Introduction	**3**	Objectives: State specific objectives, including any prespecified hypotheses.	Section 1 (Introduction), final paragraph. Three specific objectives prespecified: (1) assess the association between NSE and PCPC outcome; (2) determine an exploratory cutoff with internal validation; (3) evaluate incremental predictive value beyond PRISM III. STROBE/TRIPOD adherence stated.
Methods			

STROBE Section	Item No.	Recommendation	Location in manuscript
Methods	**4**	Study design: Present key elements of study design early in the paper.	Section 2.1 (Study Design and Ethics Approval). Single-center, retrospective, observational cohort study; setting, period, and ethics approval (Approval No: 389/2025) explicitly stated.
Methods	**5**	Setting: Describe the setting, locations, and relevant dates, including periods of recruitment, exposure, follow-up, and data collection.	Section 2.1 — Pediatric Intensive Care Unit of Gaziantep City Hospital (tertiary referral center, southeastern Turkey); admissions January 2024 to November 2025.
Methods	**6**	(a) Participants: Give the eligibility criteria, and the sources and methods of selection of participants.	Section 2.2 (Study Population). Inclusion criteria (4 items) and exclusion criteria (6 items) explicitly listed. Inclusion criterion (2) requires CSE as the primary indication for PICU admission; exclusion criterion (6) excludes PICU-acquired SE (revised in response to Reviewer 3). Patient flow described in Section 3.1 (158 screened, 26 excluded, 132 included).
Methods	**7**	Variables: Clearly define all outcomes, exposures, predictors, potential confounders, and effect modifiers. Give diagnostic criteria, if applicable.	Section 2.3 (Clinical Definitions and Classification): ILAE-based definitions of SE, RSE, and SRSE [refs 1, 19]; etiological classification [ref 20] with a priori dichotomization for multivariable analysis. Section 2.4 (Data Collection): all variables defined. Section 2.5 (Outcome Assessment): primary outcome (ΔPCPC $\geq$ 1 or in-hospital death), secondary outcomes, and PCPC scale [ref 24] defined.
Methods	**8***	Data sources/measurement: For each variable of interest, give sources of data and details of methods of assessment (measurement). Describe comparability of assessment methods if there is more than one group.	Section 2.4 (Data Collection) — itemized data fields and sources (institutional electronic health record). Serum NSE: electrochemiluminescence immunoassay, institutional upper reference limit 25.0 μg/L. PRISM III, PELOD-2, GCS scoring methods cited [refs 21, 22, 23]. Section 2.5 — PCPC scoring by two independent reviewers (M.Y., İ.B.) using standardized criteria; reviewers blinded to serum NSE values; discrepancies resolved by consensus discussion. Formal inter-rater reliability statistics were not assessed and could not be reconstructed retrospectively.
Methods	**9**	Bias: Describe any efforts to address potential sources of bias.	Section 2.5 — Outcome ascertainment bias addressed by dual independent review with consensus resolution; reviewers blinded to

STROBE Section	Item No.	Recommendation	Location in manuscript
			serum NSE values when assigning PCPC scores. Formal inter-rater reliability statistics were not assessed and could not be reconstructed retrospectively (acknowledged as a limitation in Section 4.6). Section 2.7 — confounding addressed by multivariable adjustment (Tables 3, 4); collinearity addressed by VIF analysis (max 1.41 primary, 1.72 full sensitivity); optimism addressed by bootstrap internal validation (Section 3.5).
Methods	**10**	Study size: Explain how the study size was arrived at.	Section 2.8 (Sample Size Considerations). All consecutive eligible patients in the study period included. Justification by events-per-variable rule [ref 25]: 60 events / 4 predictors = EPV 15 for the primary parsimonious model. Mortality-only model (18 events) acknowledged as exploratory and restricted to three predictors.
Methods	**11**	Quantitative variables: Explain how quantitative variables were handled in the analyses. If applicable, describe which groupings were chosen and why.	Section 2.7 (Statistical Analysis). Continuous variables tested for normality (Shapiro–Wilk) and reported as mean ± SD or median (IQR). NSE entered as continuous predictor (per 1 μg/L). Etiology dichotomized a priori (acute symptomatic vs other; rationale in Section 2.3). NSE sampling-time categorization and stratified analyses described in Section 2.7 and Section 3.6.
Methods	**12**	(a) Statistical methods: Describe all statistical methods, including those used to control for confounding.	Section 2.7 — comprehensive description: software (SPSS 26.0, MedCalc 22.0, Python 3.12 with statsmodels/scikit-learn/lifelines); tiered multivariable strategy (primary parsimonious + full sensitivity + outcome-stratified models); bootstrap optimism correction (2000 resamples, Harrell procedure [ref 26]); calibration (Hosmer–Lemeshow [ref 27], Brier, LOWESS plot [ref 28], optimism-corrected slope and intercept); ROC and DeLong comparison [ref 29]; survival analysis.
Methods	**12**	(b) Describe any methods used to examine subgroups and interactions.	Section 2.7 and Section 3.8 — exploratory subgroup analyses by etiology (acute symptomatic vs other), by EEG findings (abnormal vs normal), and by MRI findings. No formal interaction tests performed; subgroup findings reported descriptively.

STROBE Section	Item No.	Recommendation	Location in manuscript
Methods	**12**	(c) Explain how missing data were addressed.	Section 2.7 (Missing data paragraph). Variables in primary and sensitivity multivariable models (NSE, PRISM III, acute symptomatic etiology, mechanical ventilation, RSE/SRSE classification, lactate, NSE sampling time) had no missing values; complete-case analysis. S100B measurements were available in only 2/132 patients and were therefore not analyzed and not entered into multivariable models. No imputation.
Methods	**12**	(d) If applicable, explain how loss to follow-up was addressed.	Not applicable — outcome assessed at hospital discharge for all 132 included patients.
Methods	**12**	(e) Describe any sensitivity analyses.	Section 2.7 — three prespecified sensitivity strategies: (1) full sensitivity multivariable model with seven predictors (Table 3, right column); (2) restriction to NSE sampling within 24 h of SE onset (Section 3.6); (3) outcome-stratified multivariable models for mortality and survivor-only deterioration (Table 4).
Results			
Results	**13***	(a) Participants: Report numbers of individuals at each stage of the study—e.g., numbers potentially eligible, examined for eligibility, confirmed eligible, included in the study, completing follow-up, and analysed.	Section 3.1 (Patient Characteristics), first paragraph. 158 screened, 26 excluded (reasons individually itemized), 132 included and analysed. EEG performed in 105/132 (79.5%) and MRI in 92/132 (69.7%); non-standardized acquisition (Section 2.4) — reported descriptively in Section 3.1 only.
Results	**13***	(b) Give reasons for non-participation at each stage.	Section 3.1 — exclusion reasons listed: 11 missing NSE within 48 h; 7 traumatic brain injury; 4 cardiac arrest–related SE; 3 hemolyzed samples; 1 neuroblastoma.
Results	**13***	(c) Consider use of a flow diagram.	Patient flow described narratively in Section 3.1 with all exclusion reasons quantified; flow diagram considered but a narrative description was deemed sufficient given the modest number of exclusions and the relatively simple inclusion path.
Results	**14***	(a) Descriptive data: Give characteristics of study participants (e.g., demographic, clinical, social) and information on exposures and potential confounders.	Section 3.1 and Section 3.2; Table 1 (comprehensive comparison of demographic and clinical characteristics between good and poor outcome groups, including age, sex, seizure duration, SE severity, etiology,

STROBE Section	Item No.	Recommendation	Location in manuscript
			severity scores, NSE, lactate, CRP, mechanical ventilation, PICU LOS, and mortality).
Results	**14***	(b) Indicate number of participants with missing data for each variable of interest.	Section 3.1 and Table 1 footer. S100B: available in only 2/132 patients; not included in multivariable analyses. All other variables in multivariable models complete.
Results	**14***	(c) Summarise follow-up time (e.g., average and total amount).	Section 3.2 and Table 1 — PICU length of stay reported (median 5 days, IQR 4–7). Outcome assessed at hospital discharge.
Results	**15***	Outcome data: Report numbers of outcome events or summary measures over time.	Section 3.2. Primary outcome: 60/132 (45.5%) poor neurological outcome (18 deaths + 42 ΔPCPC ≥ 1 survivors). Mortality 18/132 (13.6%). NSE distribution by outcome group, mortality status, and severity category presented in Section 3.7 (Figure 4).
Results	**16**	(a) Main results: Give unadjusted estimates and, if applicable, confounder-adjusted estimates and their precision (e.g., 95% confidence interval). Make clear which confounders were adjusted for and why they were included.	Table 2 (univariate logistic regression for all candidate predictors with 95% CI and p). Table 3 (primary parsimonious and full sensitivity multivariable models, side-by-side, with 95% bootstrap percentile CI). Table 4 (mortality-only and survivors-only multivariable models, side-by-side). Rationale for covariate selection in Section 2.7 ("Multivariable analysis strategy" paragraph).
Results	**16**	(b) Report category boundaries when continuous variables were categorized.	Continuous variables retained as continuous in all multivariable analyses. NSE cutoff for ROC analysis (25.7 μg/L) determined by Youden index, presented as exploratory (Section 3.6, Table 5). SE severity categorized into SE/RSE/SRSE per ILAE definitions (Section 2.3).
Results	**16**	(c) If relevant, consider translating estimates of relative risk into absolute risk for a meaningful time period.	Section 3.7 — absolute event counts reported: 14/30 deaths above NSE cutoff vs 4/102 below (Figure 5, Kaplan–Meier). Diagnostic operating characteristics with absolute proportions and Wilson 95% CIs in Table 5.
Results	**17**	Other analyses: Report other analyses done—e.g., analyses of subgroups and interactions, and sensitivity analyses.	Section 3.5 (mortality-only and survivors-only multivariable models, Table 4); Section 3.6 (NSE sampling-time sensitivity analysis: AUC 0.763 in 113 patients with NSE measured within 24 h); Section 3.8 (subgroup analyses by etiology, EEG, and MRI findings); Figures 1 (calibration), 2 (ROC), 3 (sampling time), 4 (NSE boxplots), 5 (Kaplan–Meier).

STROBE Section	Item No.	Recommendation	Location in manuscript
Discussion			
Discussion	**18**	Key results: Summarise key results with reference to study objectives.	Section 4 (Discussion), opening paragraph. Each of the three prespecified objectives addressed: (1) NSE–outcome association established (aOR 1.11, p = 0.001); (2) exploratory cutoff at 25.7 µg/L characterized with high specificity and PPV but limited sensitivity; (3) incremental value over PRISM III shown to be modest and not statistically significant (DeLong p = 0.103).
Discussion	**19**	Limitations: Discuss limitations of the study, taking into account sources of potential bias or imprecision. Discuss both direction and magnitude of any potential bias.	Section 4.6 (Limitations) — itemized limitations: (1) retrospective single-center design; (2) NSE sampling-time variability; (3) short-term outcome only and absence of formal inter-rater reliability statistics for retrospective PCPC scoring (Pujar 2018 reference for long-term context); (4) cutoff derived and tested in same cohort (optimism); (5) PICU-acquired SE excluded; (6) no continuous EEG, no CSF NSE, no NfL/GFAP; (7) external validation needed (TRIPOD reference).
Discussion	**20**	Interpretation: Give a cautious overall interpretation of results considering objectives, limitations, multiplicity of analyses, results from similar studies, and other relevant evidence.	Section 4 (Discussion), Sections 4.1 through 4.7. Findings interpreted cautiously and explicitly framed as hypothesis-generating throughout. Comparison with prior pediatric and adult literature in Section 4.1 [refs 11–15]. New Section 4.4 addresses brain-specific injury vs systemic illness severity. Section 4.7 (Implications and Future Directions) provides a structured roadmap of required next steps.
Discussion	**21**	Generalisability: Discuss the generalisability (external validity) of the study results.	Section 4.6 (Limitations) — single-center setting in southeastern Turkey with substantial proportion of transferred patients; potential effect on severity and sampling-time distribution. Section 4.7 — prospective multicenter cohorts required for external validity. Section 5 (Conclusions) explicitly states findings are hypothesis-generating and require external validation before routine clinical use.
Other information			
Other information	**22**	Funding: Give the source of funding and the role of the funders for the present study	Back matter, Funding statement: "This research received no external funding."

STROBE Section	Item No.	Recommendation	Location in manuscript
		and, if applicable, for the original study on which the present article is based.	Conflicts of Interest statement: "The authors declare no conflicts of interest."

Notes: STROBE items marked with an asterisk (*) call for information that should be given separately for cases and controls in case-control studies and for exposed and unexposed groups in cohort and cross-sectional studies. In this single-cohort study comparing good- vs poor-outcome groups, the corresponding split is provided in Table 1 of the main manuscript.

Reference: von Elm E, Altman DG, Egger M, Pocock SJ, Gøtzsche PC, Vandenbroucke JP. The Strengthening the Reporting of Observational Studies in Epidemiology (STROBE) Statement: guidelines for reporting observational studies. Ann Intern Med. 2007;147(8):573–577.

An Explanation and Elaboration article discusses each checklist item and gives methodological background and published examples of transparent reporting: Vandenbroucke JP, von Elm E, Altman DG, et al. Ann Intern Med. 2007;147(8):W163–W194. Information on the STROBE Initiative is available at https://www.strobe-statement.org.
